# Cordycepin confers long-term neuroprotection via inhibiting neutrophil infiltration and neuroinflammation after traumatic brain injury

**DOI:** 10.1186/s12974-021-02188-x

**Published:** 2021-06-15

**Authors:** Pengju Wei, Ke Wang, Chen Luo, Yichen Huang, Dilidaer Misilimu, Huimei Wen, Peng Jin, Chuhua Li, Ye Gong, Yanqin Gao

**Affiliations:** 1 Department of Critical Care Medicine and Neurosurgery of Huashan Hospital, State Key Laboratory of Medical Neurobiology, MOE Frontiers Center for Brain Science, and Institutes of Brain Science, Fudan University, Shanghai, 200032 China; 2 School of Life Science, South China Normal University, Guangzhou, 510631 China

**Keywords:** White matter injury, Neutrophil, Microglia/macrophage, Blood-brain barrier, Traumatic brain injury

## Abstract

**Background:**

The secondary injury caused by traumatic brain injury (TBI), especially white matter injury (WMI), is highly sensitive to neuroinflammation, which further leads to unfavored long-term outcomes. Although the cross-talk between the three active events, immune cell infiltration, BBB breakdown, and proinflammatory microglial/macrophage polarization, plays a role in the vicious cycle, its mechanisms are not fully understood. It has been reported that cordycepin, an extract from *Cordyceps militaris*, can inhibit TBI-induced neuroinflammation although the long-term effects of cordycepin remain unknown. Here, we report our investigation of cordycepin’s long-term neuroprotective function and its underlying immunological mechanism.

**Methods:**

TBI mice model was established with a controlled cortical impact (CCI) method. Cordycepin was intraperitoneally administered twice daily for a week. Neurological outcomes were assessed by behavioral tests, including grid walking test, cylinder test, wire hang test, and rotarod test. Immunofluorescence staining, transmission electron microscopy, and electrophysiology recording were employed to assess histological and functional lesions. Quantitative-PCR and flow cytometry were used to detect neuroinflammation. The tracers of Sulfo-NHS-biotin and Evans blue were assessed for the blood-brain barrier (BBB) leakage. Western blot and gelatin zymography were used to analyze protein activity or expression. Neutrophil depletion in vivo was performed via using Ly6G antibody intraperitoneal injection.

**Results:**

Cordycepin administration ameliorated long-term neurological deficits and reduced neuronal tissue loss in TBI mice. Meanwhile, the long-term integrity of white matter was also preserved, which was revealed in multiple dimensions, such as morphology, histology, ultrastructure, and electrical conductivity. Cordycepin administration inhibited microglia/macrophage pro-inflammatory polarization and promoted anti-inflammatory polarization after TBI. BBB breach was attenuated by cordycepin administration at 3 days after TBI. Cordycepin suppressed the activities of MMP-2 and MMP-9 and the neutrophil infiltration at 3 days after TBI. Moreover, neutrophil depletion provided a cordycepin-like effect, and cordycepin administration united with neutrophil depletion did not show a benefit of superposition.

**Conclusions:**

The long-term neuroprotective function of cordycepin via suppressing neutrophil infiltration after TBI, thereby preserving BBB integrity and changing microglia/macrophage polarization. These findings provide significant clinical potentials to improve the quality of life for TBI patients.

**Supplementary Information:**

The online version contains supplementary material available at 10.1186/s12974-021-02188-x.

## Introduction

TBI is one of the leading causes of death and disability among all ages worldwide [1]. TBI causes multiple functional deficits in the brain, such as sensory, motor, and cognitive functions, which related to the damaged regions [2]. Primary mechanical injury triggers ionic imbalance, excitatory toxicity; calcium overload, mitochondrial dysfunction, and other secondary reactions leading to neuron death followed by neuroinflammation which can lead to additional acute and chronic brain injury [3]. White matter, which constructs 50% human brain, is mainly consisted of neurite and myelin sheaths, and it has been proved necessary for advanced brain functions [4]. Compared to gray matter (GM), WM is more susceptible to TBI [5]. WMI which includes axonal injury and myelinic degeneration is also responsible for neurological deficits in TBI [6, 7].

WMI in TBI is escalated by altered microglial/macrophage functional state and subsequent neuroinflammation [8]. After TBI, peripheral immune cells are recruited into the brain parenchyma and whereby exacerbate the breakdown of the brain-blood barrier (BBB) and promote microglia/macrophages to polarize toward a pro-inflammatory state [9, 10]. BBB disruption can simultaneously cause microglial activation in return, forming a feed-forward loop [11, 12]. In this way, three active events—immune cell infiltration, BBB disruption, and proinflammatory microglial/macrophage state—reinforce one other, creating a vicious cycle that aggravates brain injury continuously. Although the cross-talk between blood-derived immune cells and cerebral microglia plays a role in the vicious cycle, the mechanisms are not fully understood yet.

Cordycepin (also known as 3′-deoxyadenosine), a substance first isolated from *Cordyceps militaris*, has been reported for a variety of bioactivities, such as anti-cancer, anti-inflammatory, antioxidant, and antipathogenic [13]. Moreover, cordycepin also has been proved having neuroprotective effects against cerebral ischemia and hemorrhage injury [14, 15]. So far, only one article reported that cordycepin suppressed neuroinflammation and BBB leakage and reduced neurological severity scores in TBI rats within 24 h post-TBI [16]. However, whether cordycepin has long-term neuroprotective effects and the underlying mechanism are not clear.

In this study, we used a CCI-induced TBI mice model to investigate the neuroprotective effects of cordycepin and the underlying mechanism. We demonstrated that cordycepin has long-term neuroprotection against traumatic brain injury. Furthermore, we found cordycepin specifically inhibits neutrophil infiltration to ameliorate BBB disruption and pro-inflammatory microglia/macrophage polarization and consequently improve neurological function.

## Methods and materials

### Animals

Male C57BL/6 mice, 9–10 weeks old (25~28 g), were purchased from Shanghai JieSiJie Laboratory Animal Co., Ltd. Mice were housed at constant humidity and temperature with a 12-h light/dark cycle with food ad libitum. All animal experiments in the study were approved by the Animal Care and Use Committee of Shanghai Medical College, Fudan University.

### TBI model and cordycepin administration

TBI was conducted using a CCI device (TBI 0310, Precision Systems and Instrumentation). The procedure was described previously [17]. Briefly, mice were anesthetized with 3% isoflurane in 70% N_2_/30% O_2_ mixture and fixed on stereotaxic apparatus. A midline incision was made to expose the skull. An approximate 4-mm craniotomy was performed over the right parietotemporal cortex using a motorized drill. The CCI was centered a 2.5-mm lateral to the midline and a 0.5-mm anterior to bregma and was conducted with a 30-mm flat-tip impounder (velocity, 3.5 m/s; duration, 150 ms; depth, 1.5 mm). After CCI, the scalp incision was sutured, and the mice were placed on a heating pad until anesthesia recovery. Sham mice received all these procedures, except for CCI.

Cordycepin isolated from the culture medium of *Cordyceps militaris* was provided by the Guangdong Provincial Key Lab of Biotechnology for Plant Development, South China Normal University. Mice were randomly assigned to the injection of Cordycepin (dissolved in sterile saline at a concentration of 1 mg/mL) at a dose of 10 mg/kg intraperitoneally or an equal volume of sterile saline (vehicle) 2-h post-TBI and twice daily for 7 successive days. To explore the long-term protection, cordycepin was administered repeatedly in our study to ensure the efficacy according to clinical medication.

### Behavioral tests

Grid walking test, cylinder test, wire hang test, and rotarod test were carried out as previously described [18, 19].

*The grid walking test* was performed on a 40 cm×20 cm flat stainless-steel grid with a mesh size of 2 cm×2 cm 35 cm above the ground. The movement of each mouse was recorded by a camera. A foot-fault step was defined as a step that the foot did not place on the grid correctly. The data were presented as a percentage of foot-fault steps in total steps in 1 min.

*The cylinder test* was carried out in a transparent cylinder (9 cm in diameter and 38 cm in height). A camera was placed over the cylinder for 10 min recording after a mouse was placed in. Forepaw (left/right/both) touches on the wall of the cylinder were recorded from the video. The data was calculated based on the formula: left/(left+right+both)×100%.

The *wire hang test* apparatus was a 50-cm long 2mm in diameter steel wire 40cm over the ground with two platforms at each end. Mice were placed on the middle of the wire with the two forepaws and were observed for 30 s in 3~4 trials. The mice were scored according to the following criteria: 0, fell off; 1, hung onto the wire with 2 forepaws; 2, hung onto the wire with added attempts to climb onto the bar; 3, hung onto the wire with 2 forepaws and 1 or both hind paws; 4, hung onto the wire with all 4 paws and with the tail wrapped around the wire; and 5, escaped to one of the platforms.

*The rotarod test* was performed with 47650 Mouse Rota-Rod (UGO BASILE). After the mice were placed on the rods, the rods began to rotate at a speed of 5r/min and accelerated to 40r/min within 300s and went on rotating at the constant speed for 200 s (total 500 s). The latency to fall off the rotating rod was recorded. All animals were tested 3 times with a break of at least 10 min between tests. Data were presented as the mean value from 3 tests.

The same mice were used for grid walking test, cylinder test, and wire hang test before pathophysiologic studies in acute phase, and another group of mice was used for rotarod test and other long-term observations. All the behavioral tests above were pre-trained for 3 days before TBI and were performed in a blind manner.

### Immunofluorescence staining

After anesthetized deeply, the mice were perfused intracardially with ice-cold saline and 4% paraformaldehyde. The brain was removed and immersed in 4% paraformaldehyde, 20% sucrose, and 30% sucrose in sequence to complete after-fixing and dehydration. A 25-μm coronal section was collected using the freezing microtome (HM525NX, ThermoFisher).

After washed in PBS and PBS+0.3% Triton, brain sections were incubated in PBS+1% Triton to break the cell membrane and then were blocked with 10% goat serum or donkey serum for 1 h. M.O.M. Kit was used to block mouse antibody. Brain sections were incubated with primary antibodies overnight at 4°C. After washing, the sections were incubated for 2 h at room temperature with secondary antibodies conjugated with Alexa Fluor-488/594/647. DAPI-Fluoromount-G was added and covered the sections with a piece of a clear coverslip.

Ten serial sections with an interval of 11 sections were stained to calculate the volume (mm^3^) of tissue loss (= the volume of left hemisphere - volume of right hemisphere).

### Transmission electron microscopy

Fresh brain tissue (thickness <1 mm) was fixed with 2.5% glutaraldehyde at 4°C. After dehydrated in graded ethanol and acetone, the brain tissue was embedded in epoxy resin and made into 50 nm sections. The prepared ultrathin sections were stained with uranyl acetate and lead citrate and then observed under Philips CM120 transmission electron microscope and photographed. Five images obtained from each mouse were used for data statistics.

### Electrophysiology

Compound action potentials (CAPs) in the external capsule were recorded as previously described [20]. Mice were anesthetized with isoflurane and were decapitated to remove the brains rapidly. A 350-μm brain slices coronal were made using a vibratome (1200s, Leica). Slices were transferred in artificial cerebrospinal fluid (aCSF) saturated with 95% O_2_+5% CO_2_ mixture at 32°C for 0.5 h and at room temperature for 1 h subsequently for recovery. The aCSF contained 124 mM NaCl, 2.5 mM KCl, 2 mM CaCl_2_, 1 mM NaH_2_PO_4_, 24 mM NaHCO_3_, 1.3 mM MgSO_4_, and 10 mM d-glucose. A concentric stimulating electrode and a glass microelectrode (5~8 MΩ) were used for stimulating and recording. CAPs were induced by monophase square waves (0.1 ms duration) with the stimulus generator (STG 4002, Multichannel). Signals evoked were amplified by Axoclamp 700B (Molecular Devices) and digitized Axon Digidata 1440A (Molecular Devices).

### Quantitative real-time PCR (q-PCR)

Real-time PCR was performed as described [21]. Briefly, the PCR was performed with the Hieff® qPCR SYBR® Green Master Mix (No Rox) (Shanghai Yeasen, China) in Mastercycler ep realplex (Eppendorf) using corresponding primers (supplementary materials). The cycle time values of the genes of interest were first normalized with glyceraldehyde-3-phosphate dehydrogenase of the same sample and then the gene expression levels were calculated and expressed as fold.

### Flow cytometry

Blood was obtained from the heart of anesthetized mice. And then mice were perfused with cold Hank’s balanced salt solution (HBSS) to collect the brains. The brains were digested with Neural Tissue Dissociation Kit (Miltenyi) using gentleMACS Octo Dissociator with heaters (Miltenyi). Immune cells were enriched with Percoll (GE Healthcare) and Ficoll (eBioscience) from the brain and blood, respectively. Cells were blocked with rat anti-mouse CD16/32 (eBioscience) and then were incubated with eFluor 450 conjugated antibody to CD45 (eBioscience), APC-Cy7 conjugated antibody to CD11b (eBioscience), PerCP-Cy5 conjugated antibody to CD11c (eBioscience), BV605 conjugated antibody to Ly6C (BD), PE conjugated antibody to Ly6G (eBioscience), APC conjugated antibody to CD3 (eBioscience), and FITC conjugated antibody to CD19 (eBioscience). Flow cytometry was performed on Beckman CytoFlex. Data were analyzed with FlowJo software.

### BBB permeability assay

Evans blue and sulfo-NHS-biotin were used to determined BBB permeability. Briefly, mice were injected with 4% Evans blue (200 mg/kg) from the femoral vein and perfused at 2h after injection. Brains were dissected into 1-mm-thick slices. Blue areas on each slice were added up and multiplied by the thickness to calculated leakage volume.

Sulfo-NHS-biotin (7 mg/kg) was dissolved in saline and injected in the femoral vein of the mice. Mice were perfused at 1 h after injection and fixed with 4% paraformaldehyde for the freezing section. Ten serial sections the same as the preceding text were stained with streptavidin-conjugated Cy3. The volume of Sulfo-NHS-biotin leakage was obtained by accumulating the area of Sulfo-NHS-biotin in each section and multiplying by 0.3 mm.

### Chemotaxis assay

Mice were killed after anesthetized excessively to extract the bone marrow from the femurs and tibias in the clean bench, and neutrophils were isolated by immunomagnetic negative selection following the manual of EasySep™ Mouse Neutrophil Enrichment Kit (STEMCELL Technologies). Neutrophils purity was measured using flow cytometry with antibodies to CD11b and Ly6G.

As described previously [22], isolated neutrophils were seeded on the upper compartment of a 24-Transwell with 8-μm pores on the permeable supports (Costar, Corning Incorporated) at a concentration of 10^6^ cells/mL with COR (10 μg/mL). Cells were allowed to migrate for 2 h against gradients of CXCL2 (10 ng/mL, Novoprotein, Shanghai) at 37°C. Flow cytometry was used to count cells in the basal chamber to calculate the percentage of migration (migrated cells/10^6^×100).

### Western blot

Total protein was extracted with RIPA lysis buffer (Shanghai Beyotime, China) containing protease inhibitor cocktails (Roche). Equal amounts of protein were run on SDS-PAGE gels and transferred onto polyvinylidene difluoride membranes. 3% BSA was used for blocking. The membranes were incubated with primary antibodies, rabbit anti-ZO-1 (1:250 61-7300, Invitrogen), rabbit anti-MMP-2 (1:1000 ab97779, Abcam), rabbit anti-MMP-9 (1:1000 ab38898, Abcam), and overnight at 4°C, followed by incubation with horseradish peroxidase-conjugated secondary antibodies. Blots were imaged using ChemiDoc MP (Bio-Rad).

### Gelatin zymography

As described previously [23], the undenatured protein was mixed with loading buffer free of beta-mercaptoethanol and loaded on SDS-PAGE gel containing 1% gelatin. After electrophoresis, the gel was washed with 2.5% TritonX-100 and ddH_2_O and then incubated in medium (50 mM Tris, 150 mM NaCl, 10 mM CaCl2, 1% TritonX-100, pH7.5) for 42 h at 37°C. At last, the gel was stained with 0.5% Coomassie blue staining solution and was imaged using ChemiDoc MP (Bio-rad) with a white tray.

### Neutrophils depletion

As described [24], to depletion neutrophils, each mouse received twice intraperitoneal administration of 100 μg monoclonal anti-mouse Ly6G (clone 1A8, BE0075-1, BioXcell) at 24 h before TBI and 24h after TBI. Each control mouse received 100ug isotype control antibody rat IgG2a in the same manner.

### Image processing and statistical analysis

Sample sizes are determined using a sample size calculator from DSS Research, assuming an α error of 5%, a β error (1-statistical power) of 20%, and the standard deviation of data samples within each group as 25% of the mean value, based on our published studies and pilot experiments. All images were quantitated with ImageJ software. 3D images were made with Imaris 9. The data were analyzed using SPSS Statistics 20 and GraphPad Prism 8. All values are presented as mean±SE. Multiple comparisons were analyzed using one-way ANOVA and post hoc Bonferroni test. Repeated measures data were analyzed using one-way ANOVA with repeated measures and multivariate analysis of variance (MANOVA). When comparing two groups, unpaired Student’s t test was used. *p*<0.05 was considered to be statistically significant.

## Results

### Cordycepin administration ameliorates neurological deficits after TBI and reduces both GMI and WMI

**Fig. 1:**
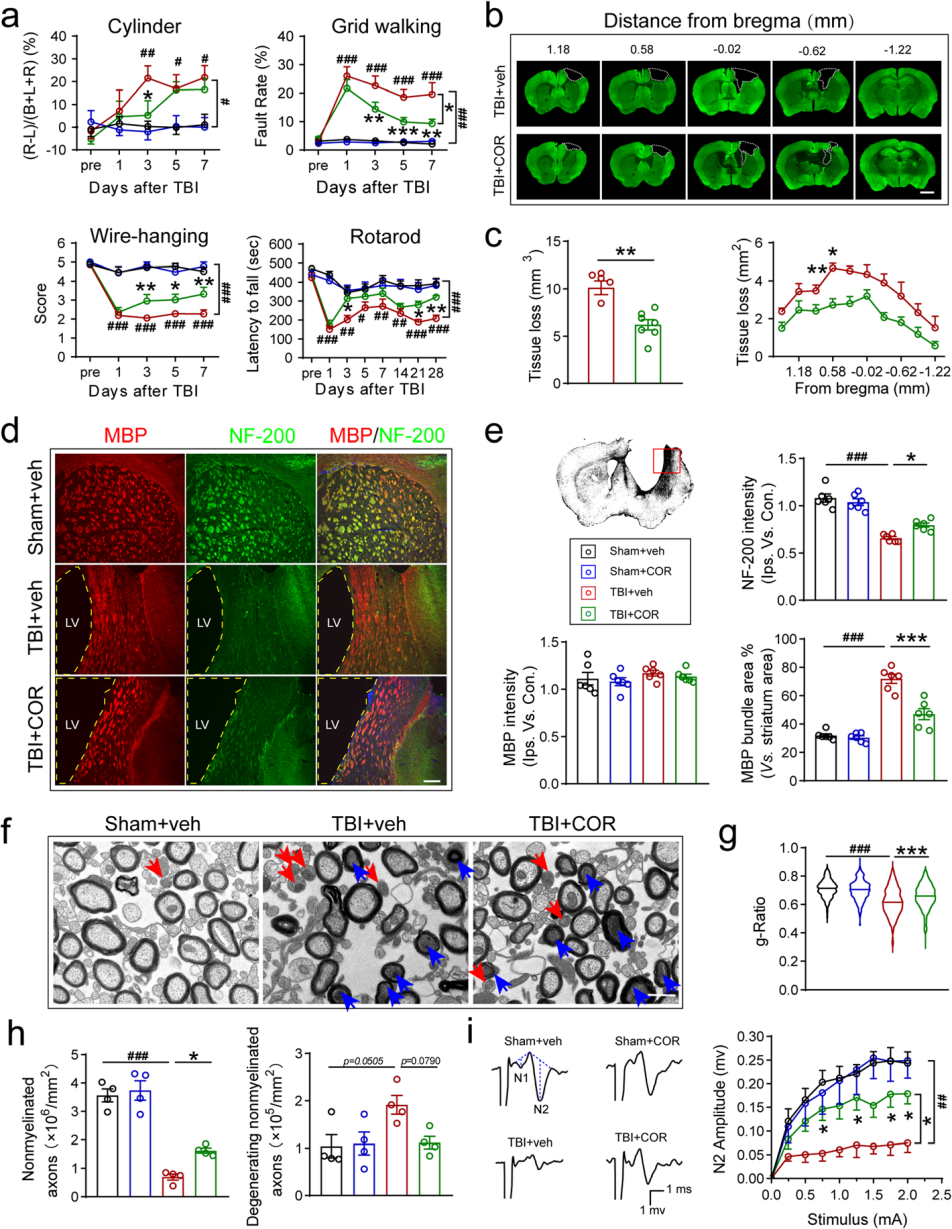
Effect of cordycepin on sensorimotor behavior, GMI and WMI. **a** Cylinder test, grid walking test, and wire-hanging test conducted for 7 days after TBI, and rotarod test up to 28 days after TBI. *n*=9/group. **b** Representative images of serial coronal sections labeled with MAP2 35 days after TBI. Scale bar: 1 mm. **c** The otal tissue loss volume and tissue loss area of each serial section. *n*=6~7/group. **d** Representative MBP/NF-200 stained images of the striatum 35 days after TBI. Scale bar: 50 μm. The location of the representative image was indicated with a red pane in the schematic picture. **e** Quantification of NF-200 fluorescence intensity, MBP fluorescence intensity, and percentage of bundles area in the striatum. *n*=6/group. **f** Representative transmission electron microscope images of the corpus callosum 35 days after TBI, blue arrows and red arrows indicate degenerating myelinated axons and nonmyelinated axons, respectively. Scale bar: 500 nm. **g** Quantification of the g-Ratio of myelinated axons (inner diameter/outside diameter). **h** Quantification of the numbers of nonmyelinated axons and degenerating nonmyelinated axons. *n*=4/group. Random 100 axons of each mouse were focused for g-Ratio statistics. **i** Representative CAPs and quantification of N2 amplitude. *n*=6/group. veh (vehicle), COR (cordycepin), ^#^*p* < 0.05, ^##^*p* < 0.01, and ^###^*p* < 0.001, or *p < 0.05, **p < 0.01, and ***p < 0.001 as indicated

Due to the high degree of spontaneous and timing-dependent functional recovery in cortical and subcortical injured rodents, it is necessary to use multiple tests for a more reliable evaluation for dynamic recovery [25]. Therefore, we employed a battery of behavioral tests (cylinder test, grid walking test, wire hanging test, and the rotarod test) to evaluate the effect of cordycepin on TBI-induced sensorimotor function defects(Fig. 1a). No obvious difference was observed between cordycepin and saline administration in sham mice. However, 1-week tests after TBI showed that cordycepin reduced forelimb asymmetry in the cylinder test, decreased foot fault rate in the grid walking test, and improved scores in the wire hanging test. Neglection on long-term outcomes of TBI is one of the challenges that prevents successful translation between clinical and pre-clinical studies [26]. Hence, rotarod test was continued to 28d after TBI for tracking long-term outcomes. Cordycepin prolonged duration on rotarod even on 21 days and 28 days after TBI, suggesting a long-term pro-recovery effect of cordycepin. These data indicated that cordycepin administration demonstrated superior sensorimotor functional recovery after TBI compared with vehicle mice.

Immunohistochemical staining of MAP2 on the serial coronary sections was conducted for assessing the neuronal tissue loss (Fig. 1b). Cordycepin significantly decreased the volume of neuronal tissue loss compared with vehicle mice (Fig. 1c).

Besides gray matter, white matter is also the main target of TBI closely linked to neurological impairment [21]. Firstly, MBP and NF-200 were stained to observe myelin and neurofilament changes in the striatum after TBI (Fig. 1d). The fluorescence intensity of NF-200 significantly reduced after TBI, whereas cordycepin increased the NF-200 fluorescence intensity significantly (Fig. 1e, up-right panel). White matter fiber tracts in the striatum were perpendicular to the coronal sections we chose, constructing bundles in the sections. Although no significant difference was found between the groups by measuring the fluorescence intensity of MBP staining (Fig. 1e, down-left panel), we found that the bundles became loose and the space among the bundles decreased in perilesional striatum after TBI. Further analysis revealed that the area proportion of MBP staining bundles in vehicle mice significantly increased because of the atrophy of striatum, while cordycepin reversed the increase (Fig. 1e, down-right panel). The data manifested the effect of cordycepin on maintaining white matter structure.

It was reported that TBI led to axonal degeneration and the myelin debris could exist for a long time [27]. So MBP staining may be difficult to reflect the factual conditions of myelin. Secondly, regions of corpus callosum (CC) were dissected from mice in all groups at 35 days after TBI to examine the ultrastructure of axon and myelin via transmission electron microscope (Fig. 1f). The number of myelinated and nonmyelinated axons was both significantly decreased by TBI, whereas only nonmyelinated axons were restrained by cordycepin significantly (Fig. S1a, Fig. 1h left panel). After TBI, no substantial variety was observed in the degenerating myelinated axon number (Fig. S1b). Cordycepin administration tendentiously abolished the TBI-induced degenerating nonmyelinated axons (Fig. 1h, right panel, *p*=0.0790). Interestingly, although no obvious changes were made in the two types of myelinated axon numbers in TBI mice, the reduction of g-ratio of myelinated axons caused by TBI was ameliorated by cordycepin (Fig. 1g). These data indicated cordycepin protecting the nonmyelinated axons in number and the myelin in structure.

CAPs were usually measured to assess the conductive capacity of white matter fibers. Due to the saltatory conduction of nervous impulses between Ranvier nodes, the nerve conductive velocity on myelinated axons is faster than nonmyelinated axons. The difference of conductive velocity can be presented on the N1 and N2 peaks in CAPs. Thirdly, mice were sacrificed 35d after TBI and sections were prepared to record CAPs in CC with electrophysiology technique (Fig. S1c). The amplitude of N2 was deceased by TBI and rescued by cordycepin (Fig. S1d, Fig. 1i), while N1 amplitude had no statistical difference between veh and cordycepin groups after TBI. The results suggested cordycepin improved nerve conductive capacity of nonmyelinated fibers.

Taken together, cordycepin administration ameliorated TBI-induced neurological deficits and brain tissue injury, including gray matter and white matter injury.

### Cordycepin administration inhibits pro-inflammatory microglia/macrophage and promotes anti-inflammatory microglia/macrophage

**Fig. 2:**
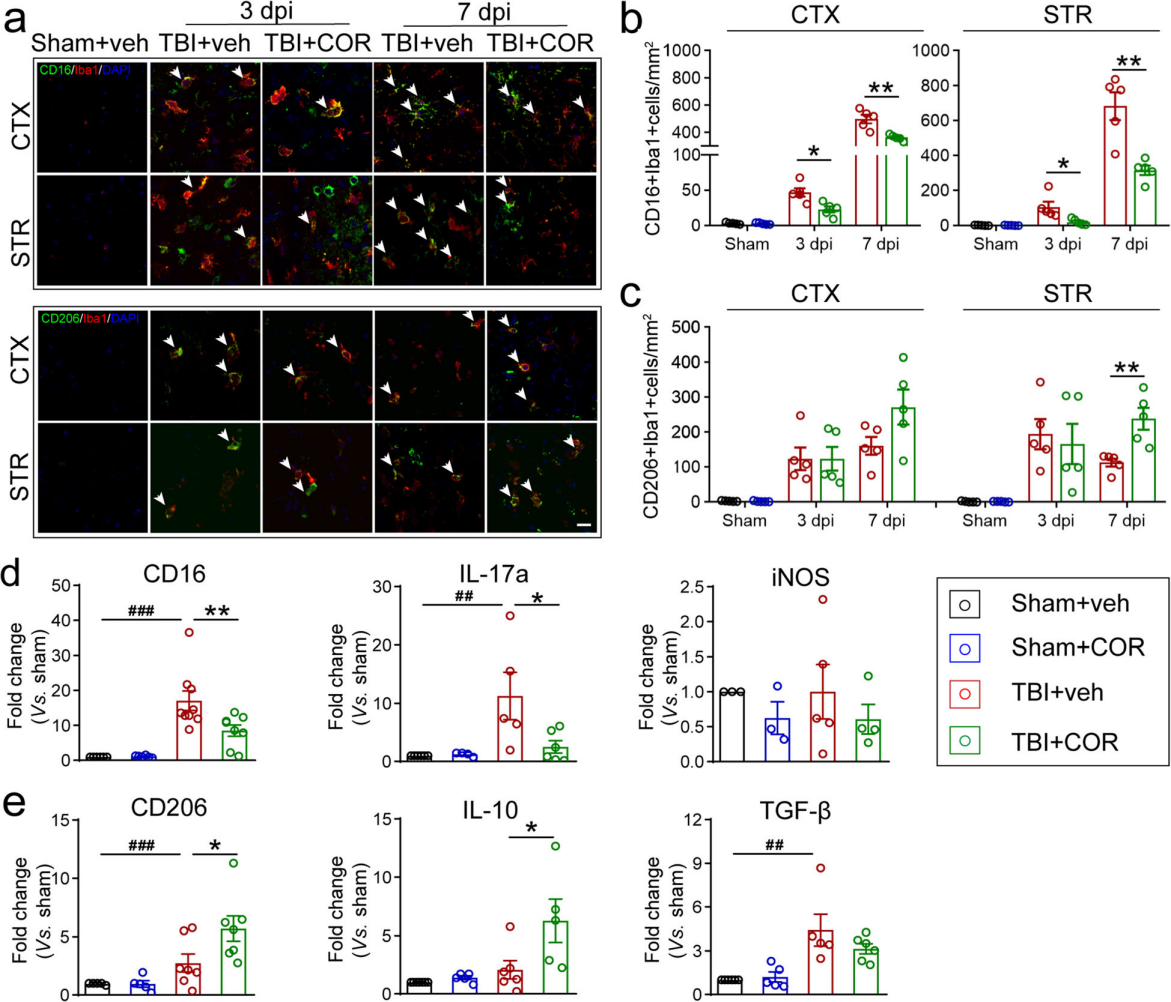
Effect of cordycepin on microglia/macrophage polarization. **a** Representative CD16/Iba1 and CD206/Iba1 stained images of cortex (CTX) and striatum (STR) on 3 days and 7 days after TBI. White arrows indicate double-positive cells. Scale bar: 20 μm. **b**, **c** Quantification of the numbers of CD16^+^Iba1^+^cells (**b**)and CD206^+^Iba1^+^cells (**c**) in CTX and STR. **d**, **e** qPCR results of pro-inflammatory (**d**) and anti-inflammatory markers (**e**) on 7 days after TBI. n=3-7/group. ^##^*p* < 0.01 and ^###^*p* < 0.001; or *p < 0.05, **p < 0.01, and ***p < 0.001 as indicated

Neuroinflammation is one of the main causes of secondary WMI in TBI. Our previous study has proved the close correlation between microglia/macrophage polarization mediated neuroinflammation and WMI [17]. According to the temporal characteristic of microglia/macrophage polarization, we double-stained CD16/Iba1 (pro-inflammatory) and CD206/Iba1 (anti-inflammatory) on 3 days and 7days after TBI (Fig. 2a). Cordycepin inhibited pro-inflammatory microglia/macrophage in the cortex and striatum on 3 days and 7 days (Fig. 2b) and promoted anti-inflammatory microglia/macrophage in the striatum on 7 days after TBI (Fig. 2c). Besides, the polarization markers were detected on 7 days after TBI using q-PCR. Markers related to pro-inflammatory microglia/macrophage such as CD16 and IL17a were significantly decreased by cordycepin (Fig. 2d). Meanwhile, markers related to anti-inflammatory microglia/macrophage such as CD206 and IL-10 were increased in the cordycepin administration group (Fig. 2e). The results demonstrated cordycepin inhibited pro-inflammatory microglia/macrophage and promoted anti-inflammatory microglia/macrophage polarization after TBI.

### Cordycepin administration protects BBB integrity after TBI

**Fig. 3:**
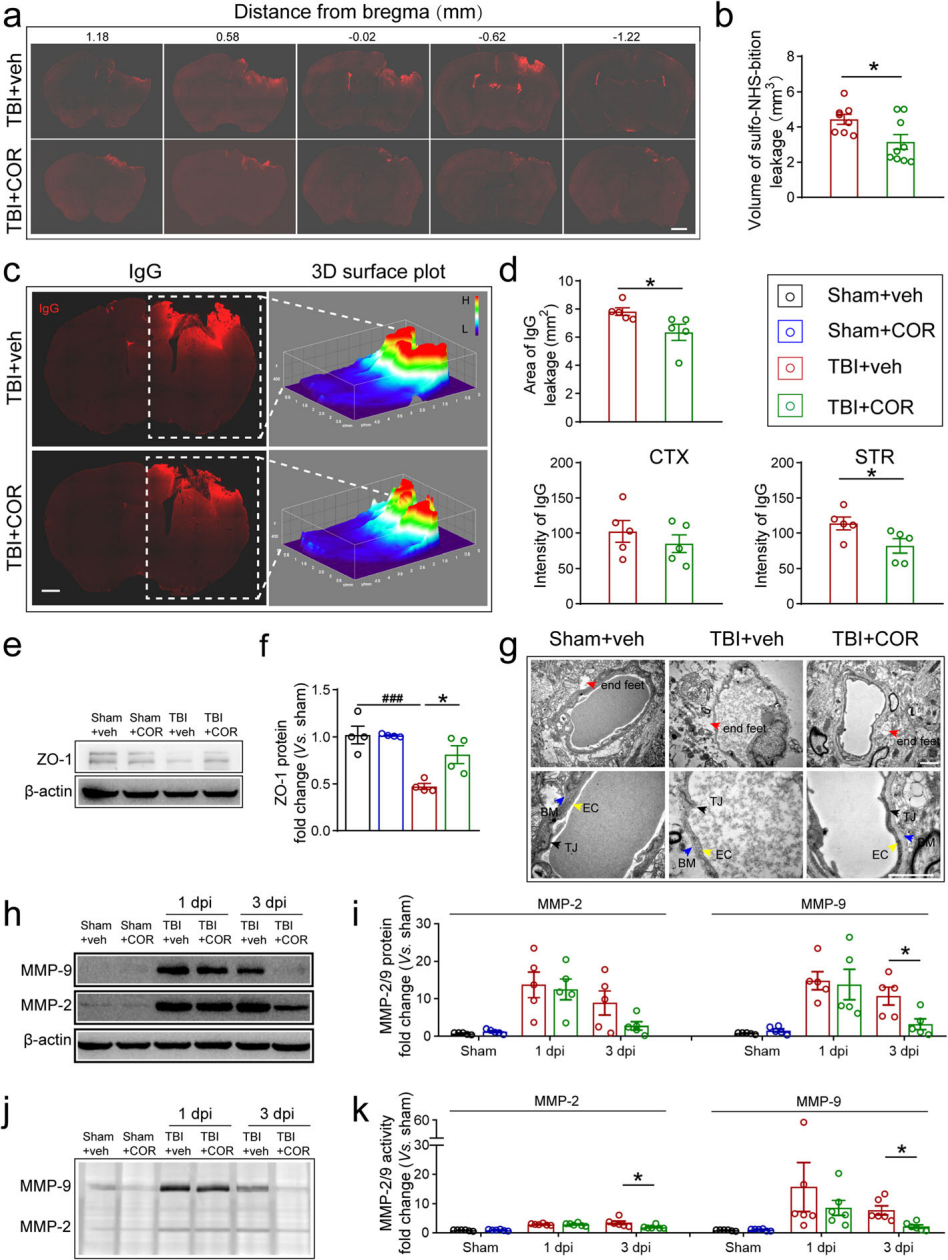
Effect of cordycepin on BBB integrity. **a** Representative images of a serial coronal sections of sulfo-NHS-biotin leakage on 3 days after TBI. Scale bar: 1 mm. **b** Quantification of sulfo-NHS-biotin leakage volume. n=7~9/group. **c** Representative images and 3D surface plot of IgG leakage on 3 days after TBI. Scale bar: 1 mm. **d** Quantification of IgG leakage area, intensity in CTX and STR. n=5/group. **e** Representative images of ZO-1 blot on 3 days after TBI. **f** Quantification of ZO-1 protein expression. n=4/group. **g** Representative transmission electron microscope images of BBB structure. Scale bar: 1 μm. **h** Representative images of MMP-2/9 blot on 24h and 3days after TBI. **i** Quantification of MM-2/9 protein expression. n=5/group. **j** Representative images of gelatin zymography. **k** Quantification of MMP-2/9 activity. n=6/group. ^###^*p* < 0.001 or *p < 0.05 as indicated

Normally, BBB prevents toxic substances in the blood from leaking into the brain parenchyma to maintain homeostasis in the brain. Preservation of BBB integrity contributes to reducing pro-inflammatory microglia/macrophage activation [28]. Therefore, we investigated the effects of cordycepin on BBB integrity in TBI mice in 3days after TBI. Firstly, the tracers of sulfo-NHS-biotin (MW 443.4 Da) and Evans blue (Evans blue-Albumin, MW 68,500 Da) [29] were injected into the femoral vein to examine the BBB permeability. Compared with TBI+vehicle group, sulfo-NHS-biotin or Evans blue leakage volume was significantly reduced by cordycepin (Fig. 3a, b, Fig. S2). The data collaboratively indicated that cordycepin reduced the leakage of both large and small molecules in TBI mice.

Endogenous IgG in the blood can leak into brain parenchyma through dysfunctional BBB, which makes IgG an important endogenous biomarker to assess the BBB leakage [30]. We stained 3-day post-TBI frozen section with an anti-mouse IgG antibody to detect the leakage of endogenous substance. IgG-positive area was remarkably reduced in the cordycepin administration group, compared with the vehicle TBI mice (Fig. 3c, d, up panel). Additionally, cordycepin reduced fluorescence intensity of IgG staining in the striatum suggesting the reduction of IgG leakage in amount (Fig. 3 days, down panel).

Tight junction proteins, such as ZO-1, are the main components of tight junctions between cerebrovascular endothelial cells supporting BBB integrity. TBI distinctly reduced ZO-1 expression detected by western blot, whereas cordycepin reduced ZO-1 protein loss after TBI (Fig. 3e–f). We also observed the ultrastructure of BBB with a transmission electron microscope (Fig. 3g). TBI led to edema in astrocyte end-feet, basement membrane thickening, and loss in tight junction proteins, while cordycepin reversed these degenerations.

MMPs are responsible for tight junction protein degradation in brain injury. Our previous study showed knocking out or pharmacological inhibition for MMPs reduced BBB permeability after brain injury [31]. MMP-2 and MMP-9 are the main two MMPs involved in BBB disorder, and they mediate early reversible and late irreversible BBB dysfunction respectively [32]. Accordingly, we observed the effects of cordycepin on MMP-9 and MMP-2 at 24 h and on 3 days after TBI. Western blot showed that cordycepin significantly reduced the protein level of MMP-9 in 3 days after TBI, but not MMP-2 (Fig. 3h, i). Many factors participate in MMPs post-transcriptional control. For example, endogenous tissue inhibitors of metalloproteinases (TIMPs) inhibit MMPs activity by high-affinity binding to the catalytic structural domain of MMPs in a non-selective manner [33]. Consequently, it is necessary to pay attention to the activity when evaluating the effects of MMPs. Based on the properties of MMP-9 and MMP-2 to catalytic degrade gelatin, we employed gelatin zymography to assess their activity. Zymography results showed that the activity of MMP-9 and MMP-2 was both reduced by cordycepin in 3days after TBI (Fig. 3j, k).

### Cordycepin administration inhibits neutrophil infiltration, without affecting the peripheral immune system

**Fig. 4:**
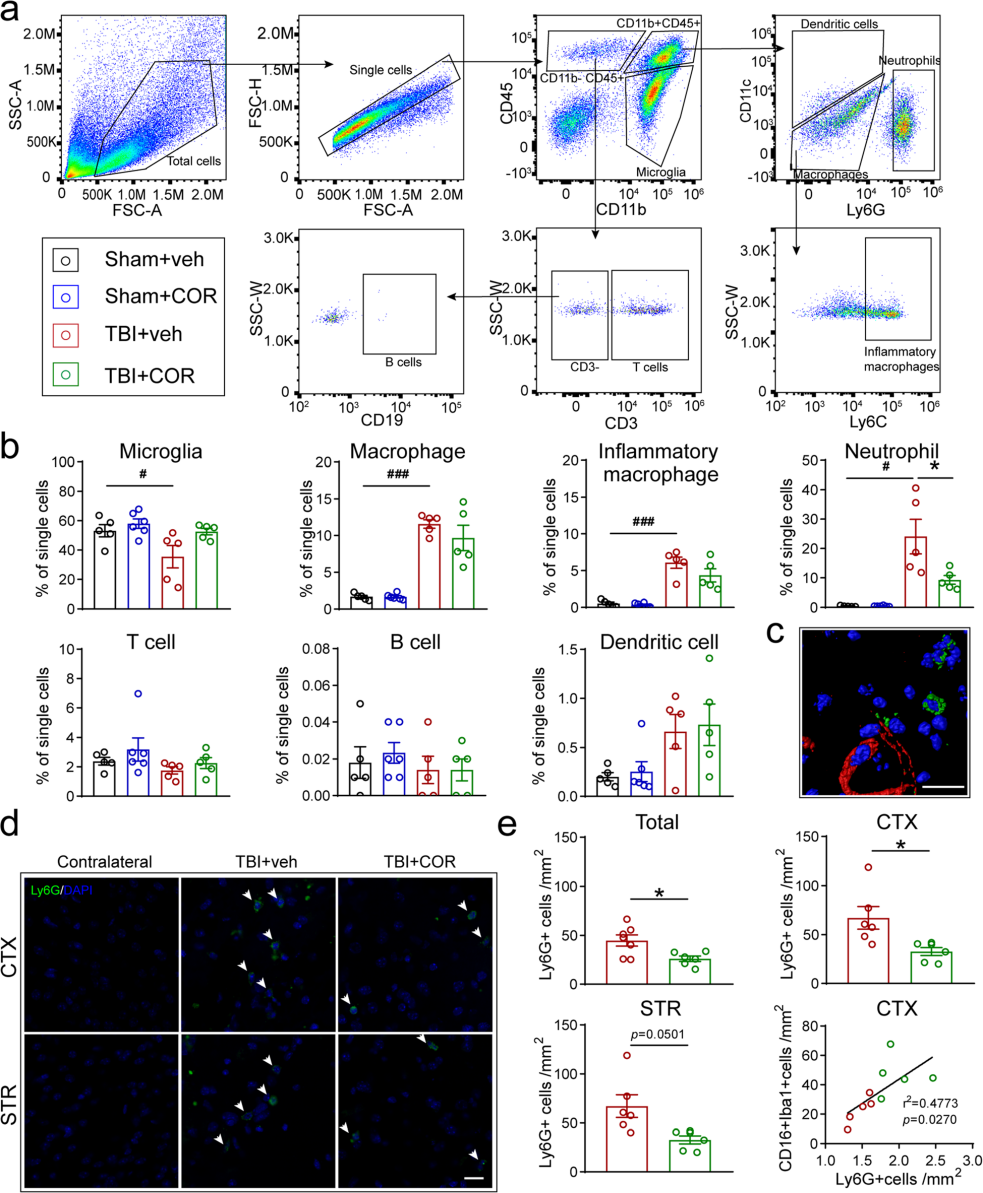
Effect of cordycepin on peripheral immune cell infiltration. **a** Illustration of flow cytometry analysis of circulating immune cells in the brain on 3 days after TBI. **b** Quantification of the numbers of infiltrating immune cells to the brain. n=5/group. **c** 3D-reconstructed image of confocal pictures of Ly6G/tomato-lectin straining. Scale bar: 20 μm. **d** Representative images of Ly6G straining. White arrows indicate Ly6G+cells. Scale bar: 20 μm. **e** Quantification of numbers of total Ly6G+cells and Ly6G+cells in CTX and STR, n=6/group. And correlation analysis of Ly6G+cells and CD16+Iba1+cells. n=5/group. ^#^*p* < 0.05, ^###^*p* < 0.001, or *p < 0.05 as indicated

The massive influx of circulating leukocytes is the biggest contributors to MMPs surging in the early stage of brain injury, especially for neutrophil, which has been proved to be the main source of MMP-9 [34, 35]. The main kinds of immune cells, including microglia, macrophage, and its inflammatory subset, neutrophil, T cell, B cell, and dendritic cell, were analyzed in the brain 3days after TBI by flow cytometry (Fig. 4a). The proportion of microglia in total cells decreased, while the proportions of macrophage, inflammatory macrophage, and neutrophil increased after TBI. Cordycepin made no significant differences in the amounts of microglia, macrophage, and inflammatory macrophage in TBI mice. However, the proportion of neutrophils was decreased by cordycepin administration. The proportion of T cell, B cell, and dendritic cell did not change obviously in each group (Fig. 4b).

Furthermore, immunofluorescent staining of Ly6G showed that the neutrophils adhered to the blood vessels or went deep in the brain parenchyma indicating that neutrophils did infiltrate after TBI (Fig. 4c). Most infiltrated neutrophils surrounded the lesion, whereas few were found in other places (Fig. 4d). The number of neutrophils was remarkably reduced by cordycepin in the total or only the cortex (Fig. 4e, up panel) and had a downtrend in the striatum (Fig. 4e, down-left panel, *p*=0.0501). More interestingly, we noticed that the number of neutrophils was significantly associated with the number of pro-inflammatory microglia/macrophage (Fig. 4e, down-right panel) suggesting that infiltrated neutrophils might promote microglia/macrophage polarizing to pro-inflammatory type.

**Fig. 5:**
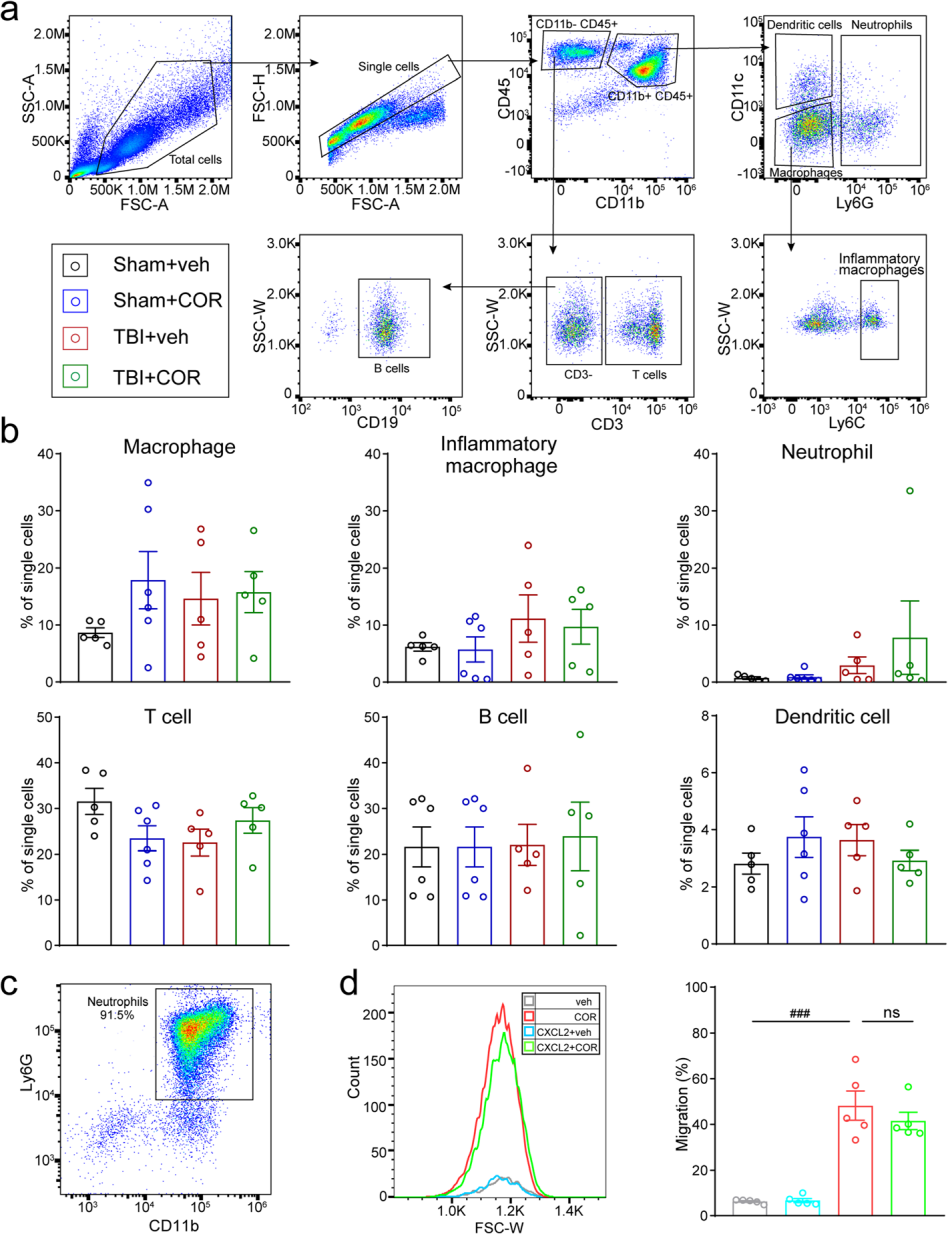
Effect of cordycepin on immune cells. **a** Illustration of flow cytometry analysis of immune cells in the blood on 3 days after TBI. **b** Quantification of numbers of immune cells in the blood. n=5/group. **c** The purity of neutrophils isolating from bone marrow (Ly6G^+^). **d** Representative histogram of flow cytometry for cell counting (left panel) and quantification of the percentage of neutrophils migration to the basal chamber. n=5/group. ^###^*p* < 0.001, ns: no significance, as indicated

The immune cells in the blood were also detected by flow cytometry (Fig. 5a). However, macrophages, inflammatory macrophages, neutrophils, T cells, B cells, and dendritic cells in the blood were not significantly affected by TBI or cordycepin at 3 days after TBI. The results indicated cordycepin did not aggravate immunosuppression induced by brain injury obviously. Moreover, its inhibition of neutrophils was more likely due to the chemotaxis in CNS. To verify the suppose, we employed a transwell assay to determine the effect of cordycepin on neutrophil chemotaxis. CXCL2 was added in the culture medium providing chemotaxis. The purity of neutrophils isolated from the bone marrow was 91.5% (Fig. 5c). CXCL2 exhibited strong chemotaxis toward neutrophils, which drew about half of the neutrophils into the basal chamber (Fig. 5d), whereas cordycepin did not affect neutrophil chemotaxis, indicating that cordycepin does not act on neutrophils in a direct manner.

### Inhibiting neutrophil infiltration by cordycepin ameliorates BBB disruption and microglia/macrophage pro-inflammatory polarization

**Fig. 6:**
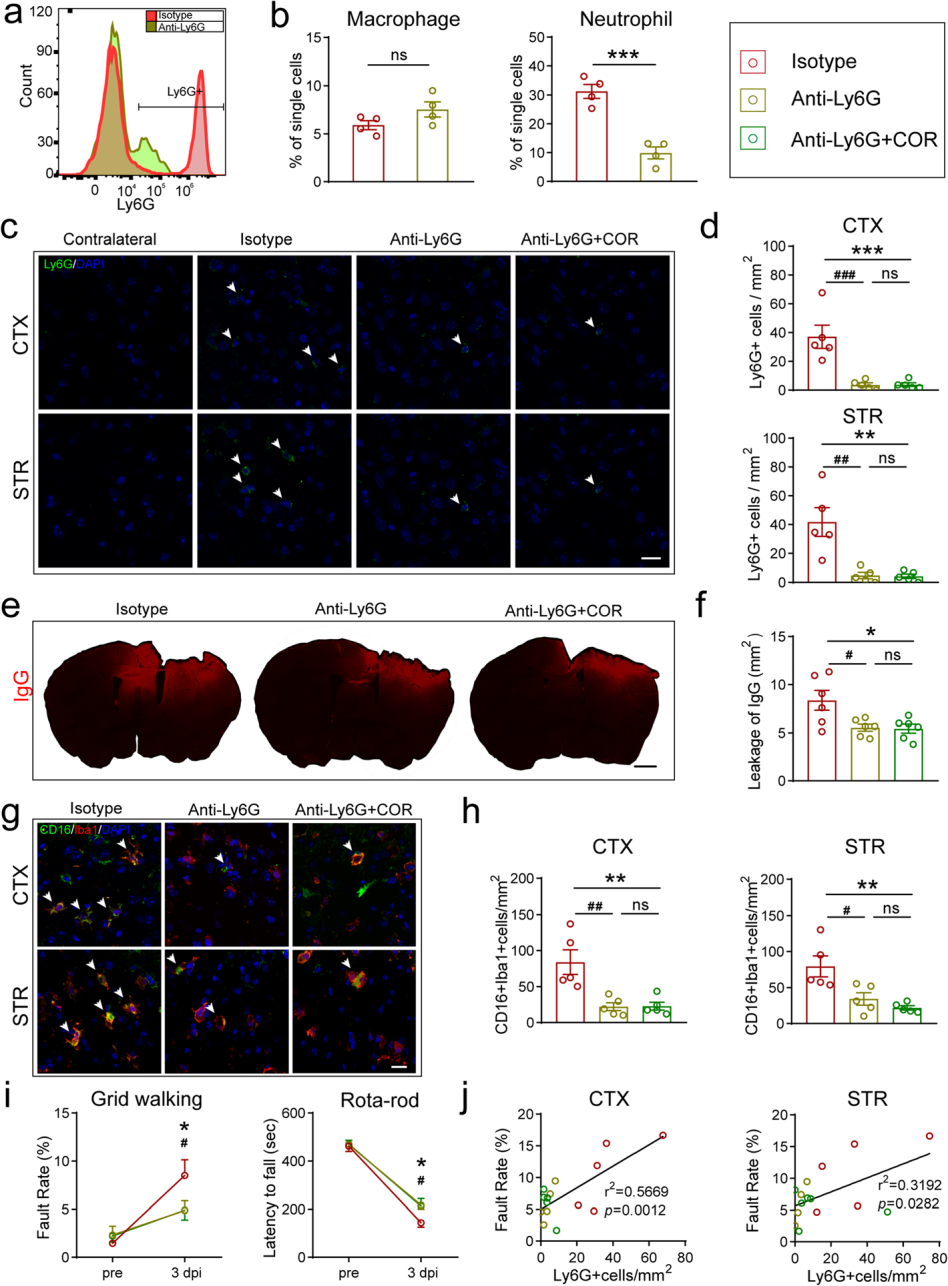
Neutrophil depletion abolished cordycepin conferred protection. **a** Representative images of flow cytometry analysis of neutrophils (Ly6G+) in the blood on 3 days after TBI. **b** Quantification of numbers of macrophages or neutrophils in the blood. n=4/group. **c** Representative images of Ly6G staining, white arrows indicate Ly6G+cells. Scale bar: 20 μm. **d** Quantification of numbers of Ly6G+cells in CTX and STR. *n*=5/group. **e** Representative images of IgG leakage on 3 days after TBI. Scale bar: 1 mm. **f** Quantification of IgG leakage area. *n*=6/group. **g** Representative CD16/Iba1 stained images in CTX and STR on 3 days after TBI, white arrows indicate CD16+Iba1+ cells. Scale bar: 20 μm. **h** Quantification of numbers of CD16+Iba1+ cells in CTX and STR. *n*=5/group. **i** Rotarod test and grid walking test results on 3 days after TBI. *n*=7~9/group. **j** Foot fault rate in grid walking test was correlated with the number of infiltration neutrophils (Ly6G^+^) in the brain. *n*=5/group. ^#^*p* < 0.05, ^##^*p* < 0.01, and ^###^*p* < 0.001 vs*.* isotype group, *p < 0.05, **p < 0.01, and ***p < 0.001 vs*.* anti-Ly6G group, ns: no significance, as indicated

To investigate the relationships among three events (neutrophil infiltration, BBB disruption, and microglia/macrophage polarization), anti-Ly6G monoclonal antibody (anti-Ly6G) was intraperitoneally injected twice for neutrophil depletion at 24h pre- and post-TBI. By flow cytometry analyzing the blood, anti-Ly6G specifically depleted neutrophils without significant effect on macrophages (Fig. S3, Fig. 6a-b). Neutrophil depletion remarkably reduced neutrophils (Fig. 6c, d), IgG leakage (Fig. 6e, f), pro-inflammatory microglia/macrophage (Fig. 6g, h) in the brain, while cordycepin combined with neutrophil depletion did not exert a further influence on them (Fig. 6c–h). Moreover, neutrophil depletion significantly reduced the foot-fault rate on the grid test and prolonged the duration on the rotarod at 3 days after TBI, whereas the neutrophil depletion and cordycepin-combined groups did not show a further effect on neurological deficits (Fig. 6i). Interestingly, the foot fault rate showed a significant positive correlation with neutrophil in both the cortex and the striatum (Fig. 6j).

These data manifested that neutrophil infiltration contributed to BBB disruption and microglia/macrophage pro-inflammatory polarization. Neutrophil infiltration might have an upstream position among the three events in the effect pathway of cordycepin.

### Cordycepin inhibits the expression of inflammatory mediators after TBI

**Fig. 7:**
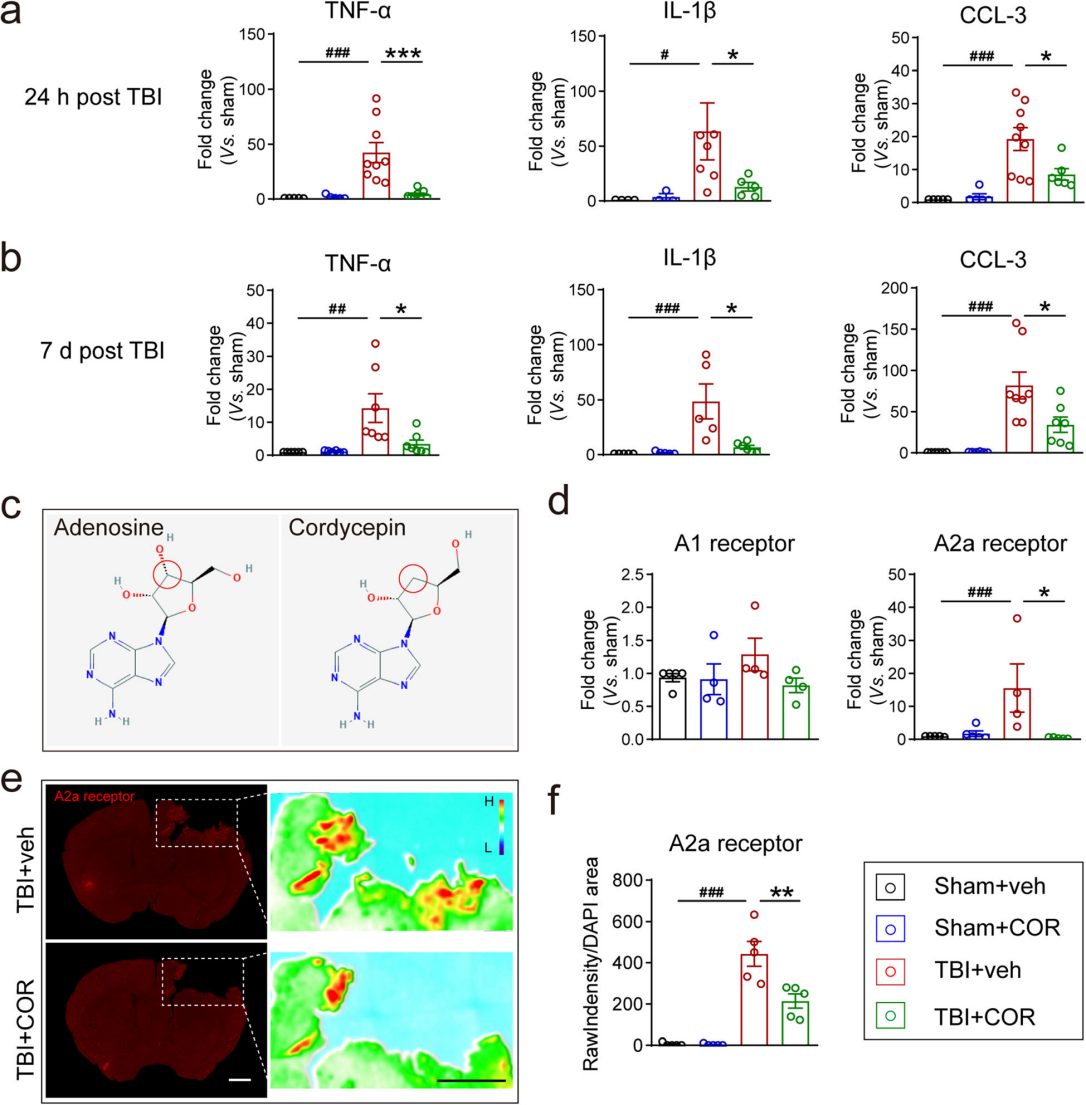
Cordycepin enhances adenosine receptors expression and ameliorates inflammatory cytokines expression after TBI. **a**, **b** Quantitative RT-PCR analysis cytokines on 3 days (**a**) and 7 days (**b**) after TBI. n=5~9/group. **c** Chemical structure of adenosine and cordycepin (from sigma website), the red circles indicate the difference between them. **d** Quantitative RT-PCR analysis of A1 and A2a receptors. *n*=4~5/group. **e** Representative images of A2a receptor expression and their heat maps. Scale bar: 1 mm. **f** Quantification of A2a receptor intensity surrounding the lesion. n=5/group. ^**#**^*p* < 0.05, ^**##**^*p* < 0.01, and ^**###**^*p* < 0.001, or *p < 0.05, **p < 0.01, and ***p < 0.001 as indicated

As the results described above, neutrophil was proved to be involved in the neuroprotection of cordycepin through depletion; meanwhile, cordycepin does not directly act on neutrophils. Then, we went back into CNS to find the answer. And we found the expression of TNF-α, IL-1β, and CCL3 mRNA in TBI mice was significantly reduced by cordycepin treatment at 24h (Fig. 7a) which is a key timepoint for neutrophil infiltration in the rise period and even 7 days (Fig. 7b). The decrease of inflammatory mediator expression in CNS is conducive to reducing the mobilization and chemotaxis for neutrophils.

Due to the remarkably similar molecular structure to adenosine (Fig. 7c), cordycepin was considered to probably act on adenosine receptors. And our previous work has proved that cordycepin exhibits biological activities through adenosine A1 and A2a receptors in CNS [36–38]. Firstly, we examined their mRNAs by qPCR at 24 h after TBI. A1 receptor mRNA expression had no substantial difference between each group (Fig. 7d, left panel), but TBI remarkably upregulated mRNA of A2a receptor, which was inhibited by cordycepin (Fig. 7d, right panel). The expression of A2a receptor at 24 h after TBI showed a consistent phenomenon by immunofluorescence staining (Fig. 7e, f). These data suggested that cordycepin might work through inhibiting A2a receptor.

## Discussion

TBI, a common disease in departments of neurology, often leads to disability or even threats to lives. In our study, we investigated the anti-inflammatory properties of cordycepin on TBI mice. Our data showed that cordycepin ameliorated neurological deficits and reduced neuronal damage and WMI, exhibiting long-term neuroprotection. Pro-inflammatory microglia/macrophage polarization was inhibited while anti-inflammatory microglia/macrophage polarization was promoted in TBI mice administered with cordycepin. Cordycepin also attenuated neutrophil infiltration and BBB disruption. Although there are complex interrelationships among microglia/macrophage polarization, neutrophil infiltration, and BBB breakdown, neutrophil depletion indicated cordycepin was specific to neutrophil infiltration. We also observed that cordycepin did not affect neutrophil chemotaxis directly, whereas cytokines and chemokines in injured brain tissue were suppressed by cordycepin treatment, suggesting that cordycepin might inhibit neutrophil infiltration through downregulating cytokines and chemokines in CNS. Cordycepin reversed TBI-induced A2a receptor high expression hinting a connection between A2a receptor and cordycepin’ effects in TBI mice.

An important reason for many promising neuroprotective drugs failed in the clinic was attributed to insufficient functional neurological tests in animal models [39]. Additionally, the inability of animals to communicate leads to the difficulty to accurately translate certain animal behaviors into symptoms or side-effects. Multiple behavior tests at different time points throughout recovery can help us understand the dynamic intervention effect more precisely. However, there are potential carryover effects of repeating behavior tests (primarily novel compensatory behavioral strategies of animals and movement rehabilitation), which may mask the deficits and true recovery [40]. We therefore used short-term grid walking test, cylinder test, and wire hang test which are little altered by repeated testing and sensitive to sensorimotor defects in another group of mice as supplements for long-term rotarod test. TBI has complicated secondary injury pathways which cause long-term progressive brain jury even lasting for years [41]. Therefore, long-term neuroprotection of a candidate drug is essential for clinical translation. In addition to short-term behaviors, we observed long-term behaviors and neuronal tissue loss, providing long-term neuroprotection evidences for cordycepin. TBI-induced WMI is positively correlated with neurological deficits, reducing WMI helps improve neurological functions [21]. We observed that cordycepin reserved the morphology of white matter and neurofilament on 35 days after TBI. MBP expression did not change at the statistical level; however, it was consistent with the previous finding that the residual myelin debris can exist for a long time after axonal fracture and degeneration caused by TBI [27]. We solidified the results by observing the ultrastructure of white matter using a transmission electron microscope which provided evidence for cordycepin’s ameliorations on axon and myelin damage. Moreover, CAPs recording offered proofs for functional improvement of white matter. Our data not only manifested the long-term neuroprotection of cordycepin but also illustrated the protective pathway of white matter.

Neuroinflammation is an important cause of secondary injury in TBI, especially for secondary WMI. Microglia is the resident immune cell in CNS, which plays a central role in CNS inflammation. The infiltrated macrophages have functional overlap with microglia post TBI [42]. Classical activated microglia/macrophage produces pro-inflammatory factors presenting pro-inflammatory function that promotes tissue damage and hinders damaged tissue remodeling, while alternative activated microglia/macrophage clears up cell debris and produces anti-inflammatory factors presenting anti-inflammatory function that promoting tissue repair [8]. Microglia/macrophage functional polarization is a promising target of pharmacological intervention in TBI. We observed that pro-inflammatory microglia/macrophage had a huge surge in cell number from 3 to 7 days after TBI, while anti-inflammatory microglia/macrophage had minute fluctuation between these days. Besides, the pro-inflammatory microglia/macrophage were far more than anti-inflammatory microglia/macrophage on 7 days after TBI. These observations were consistent with the findings of Wang et al. generally [17], indicating that although endogenous repair mechanism had been activated, it was finally defeated by pro-inflammatory microglia/macrophage over time, resulting in out-of-control neuroinflammation. Cordycepin administration significantly reduced pro-inflammatory microglia/macrophage at both 3 and 7 days after TBI and increased anti-inflammatory microglia/macrophage at 7 days after TBI. The expressions of their markers corroborated cordycepin’s effect on microglia/macrophage. The data provided confirmed evidence for the anti-inflammatory properties of cordycepin in TBI.

Under normal conditions, BBB blocks the interference of blood cells and substances for CNS. When BBB is damaged, peripheral immune cells and toxic substances leak into the brain parenchyma that can further exacerbate neuroinflammation, including microglia/macrophage pro-inflammatory polarization [11, 28]. We found that cordycepin reduced BBB leakage by injecting tracers into the femoral vein and labeling endogenous IgG. Tight junction protein ZO-1 expression was upregulated in TBI mice treated with cordycepin that provided a foundation for cordycepin protecting the structure and function integrity of BBB. MMPs degrade vascular ECM proteins and tight junction proteins, leading to BBB breakdown under pathological conditions. MMP-2 and MMP-9 are the main two MMPs involved in BBB breakdown [32]. Cordycepin decreased MMP-9 expression and inhibited the activity of MMP-2 and MMP-9, suggesting cordycepin protected BBB via the MMP-2/9 pathway. More interestingly, neutrophils are the main source of MMP-9 in acute brain injury [34]. So, we paid our attention to neutrophil, as expected, neutrophil infiltration was inhibited significantly by cordycepin administration. And we found neutrophil infiltration related to microglia/macrophage pro-inflammatory polarization. But up to this point, all of what we have seen was mere phenomena. There were no proofs to illustrate the relationships among microglia/macrophage polarization, BBB breakdown, and neutrophil infiltration. Therefore, we explored their relationships by neutrophil depletion. Neutrophil depletion exhibited cordycepin-like effects, such as reducing neutrophil infiltration, decreasing BBB leakage, inhibiting microglia/macrophage pro-inflammation polarization, and improving neurological deficits. Moreover, cordycepin administration combined with neutrophil depletion did not show additive effects. These results indicated that cordycepin was specifically effective on neutrophil infiltration, and neutrophil infiltration was upstream to BBB breakdown and microglia/macrophage pro-inflammation polarization. Circulating neutrophils are the main infiltrated peripheral immune cells within 1 h and peaking by 24 h post-injury, initiating enhanced inflammatory signals [43]. Precedence in time and neutrophil-produced MMPs mentioned above might be the reasons why neutrophils could be the point to break the vicious cycle of the three events. Our study also indicated that neutrophil infiltration might be a target to intervene TBI-induced inflammatory cascade reaction.

The analysis of immune cells in the blood showed no obvious difference made by cordycepin administration. The results suggested that cordycepin did not disturb systematic immunity and, on the other hand, inhibited neutrophil infiltration by decreasing CNS chemotaxis rather than reduced neutrophils in the blood. And our chemotaxis assay results proved cordycepin did act on neutrophils in a direct manner. Thus, we went back to CNS to find the reason for neutrophil inhibition. Cordycepin inhibited TNF-α, IL-1β, and CCL3 upregulations in CNS after TBI. CCL3 recruits neutrophils through binding to the CCR1 on the neutrophil surface, neutrophil recruitment in mice with CCL3 deficiency significantly decreased as reported [44]. Moreover, TNF-α and IL-1β mobilize neutrophils and induce adhesion molecule expression on cerebral endothelial and glial cells, thereby promoting neutrophil accumulation and migration into CNS [45–47]. We examined the expressions of the two adenosine receptors after TBI, and we observed that cordycepin reversed TBI-induced high expression of A2a receptor. Our previous work was shown that cordycepin probably acts as an antagonist to A2a receptor, since the agonist of A2a receptor (CGS 21680) rather than the antagonist (SCH 58261) could reverse the effects of cordycepin [36]. A2a receptor has been proved to promote neuroinflammation induced after TBI with A2a receptor KO mice [48]. Inhibition on A2a receptor helps reduce TBI-induced inflammation.

Our study proved long-term neuroprotection of cordycepin for TBI mice and illustrated its anti-inflammatory effects via inhibiting neutrophil infiltration to ameliorate microglia/macrophage pro-inflammation polarization and BBB breakdown. Nevertheless, there are some limitations in our study, such as how infiltrated neutrophil affects microglia/macrophage pro-inflammation polarization, and what is the detailed A2a receptor pathway of cordycepin. For the next-step work, we will focus on molecular mechanisms in cells.

Finally, there were some potential side effects of cordycepin treatments. It has been reported that cordycepin strongly inhibits protein synthesis by reducing the length of poly(A) tails and AMPK-mediated inhibition of mTOR signaling, suggesting the adverse effects on normal cellular physiology [49]. But, interestingly, cordycepin is only toxic to malignant cancer cells and has little cytotoxicity on healthy cells under safe dose, exhibiting outstanding in many pre-clinical studies [50]. Our previous work had investigated three doses at 5, 10, and 20 mg/kg by oral administration twice daily for 21 consecutive days, and the survival rate only significantly decreased in mice treated with 20 mg/kg cordycepin [14]. In toxicity and maximum tolerated dose test, cordycepin administered at a dose of 3600 mg/kg by intraperitoneal injection did not show any adverse effects on the liver, kidney, spleen, and heart weight within 14 days [51]. The dose and the duration of cordycepin used in our study are safe for mice, and no obvious side effect was observed in the study. Cordycepin is also reported suppressing peripheral inflammation through a variety of mechanisms, such as inhibitions on T cell proliferation and macrophage infiltration [52, 53]. These conditions remind us that cordycepin potentially aggravates peripheral immunosuppression, which may increase the incidence of infection in TBI patients. Our detections on peripheral immune cells suggested that cordycepin did not influence peripheral immune cells perhaps via the different immune mechanisms in our TBI model.

## Conclusion

We proved that the long-term neuroprotective function of cordycepin via inhibiting neutrophil infiltration in the acute phase is thus preserving BBB and inhibiting microglia/macrophage pro-inflammation polarization after TBI. These findings provide a basis for cordycepin clinical translation in the future.

## Supplementary Information

**Additional file1: Supplementary Fig. S1.** Effect of cordycepin on myelinated axons and CAPs N1 peak. a Quantification of the numbers of myelinated axons. b Quantification of the numbers of the degenerating nonmyelinated axons. *n*=4/group. c The locations where recording electrode and stimulus electrode were placed. d Quantification of N1 amplitude for each group. *n*=6/group. #*p* < 0.05 , as indicated. **Supplementary Fig. S2.** Effect of cordycepin on Evans blue leakage. a Representative leakage images of each group on 3d after TBI, scale bar. 1 mm. b Quantification of Evans blue leakage volume. *p < 0.05, as indicated. **Supplementary Fig. S3.** Representative Flow Cytometry analysis of the neutrophil depletion effect in blood. Blood was obtained from heart at 48 h after the second injection of Ly6G antibody. Enriched immune cells were stained with anti-Ly6C and anti-Ly6G to distinguish macrophage (Ly6C^high^Ly6G^-^) and neutrophil (Ly6C^high^Ly6G^+^).

## Data Availability

The datasets used and/or analyzed during the present study are available from the corresponding author upon reasonable request.

## Abbreviations

aCSF: Artificial cerebrospinal fluid
BBB: Brain-blood barrier
CAPs: Compound action potentials
CC: Corpus callosum
CCI: Controlled cortical impact
CCL: Chemotactic cytokine ligand
CCR: Chemotactic cytokine receptor
CD: Cluster of differentiation
CNS: Central nervous system
Cor: Cordycepin
dpi: Days post injury
ECM: Extra cellular matrix
GMI: Grey matter injury
HBSS: Hank’s balanced salt solution
IL: Interleukin
LV: Lateral ventricle
iNOS: Inducible nitric oxide synthase
MAP 2: Microtubule-associated protein 2
MBP: Myelin basic protein
WMI: White matter injury
MMPs: Matrix metalloproteinases
MW: Molecular weight
NF-200: Neurofilament-200
PBS: Phosphate-buffered saline
q-PCR: Quantitative real-time PCR
TBI: Traumatic brain injury
TGF: Transforming growth factor
TIMPs: Endogenous tissue inhibitors of metalloproteinases
TNF: Tumor necrosis factor
veh: Vehcle
ZO-1: Zonula occludens-1
