# Supplementary material for: Cordycepin confers long-term neuroprotection via inhibiting neutrophil infiltration and neuroinflammation after traumatic brain injury
Source: J Neuroinflammation. 2021 Jun 15;18:137. doi: 10.1186/s12974-021-02188-x (PMC8207641; doi:10.1186/s12974-021-02188-x)
Supplement: Supplementary file 1 — Additional file1: Supplementary Fig. S1. Effect of cordycepin on myelinated axons and CAPs N1 peak. a Quantification of the numbers of myelinated axons. b Quantification of the numbers of the degenerating nonmyelinated axons. n=4/group. c The locations where recording electrode and stimulus electrode were placed. d Quantification of N1 amplitude for each group. n=6/group. #p < 0.05 , as indicated. Supplementary Fig. S2. Effect of cordycepin on Evans blue leakage. a Representative leakage images of each group on 3d after TBI, scale bar. 1 mm. b Quantification of Evans blue leakage volume. *p < 0.05, as indicated. Supplementary Fig. S3. Representative Flow Cytometry analysis of the neutrophil depletion effect in blood. Blood was obtained from heart at 48 h after the second injection of Ly6G antibody. Enriched immune cells were stained with anti-Ly6C and anti-Ly6G to distinguish macrophage (Ly6ChighLy6G-) and neutrophil (Ly6ChighLy6G+). [file 12974_2021_2188_MOESM1_ESM.docx]

Supplementary Materials:

**Antibodies for Immunofluorescence staining**

, rabbit anti-MAP2 (1:1000, ab178846, Abcam),

rabbit anti-GFAP (1:1000, ab7260, Abcam),

rabbit anti-NF200 (1:1500, ab82259, Abcam),

rat anti-MBP (1:1500, ab40390, Abcam),

rabbit anti-Iba1 (1:1000, ab5076, Abcam),

rat anti-CD16/32 (1:250, ab25235, Abcam),

goat anti-CD206 (1:250, AF2535, R&D),

rabbit anti-CD34 (1:500, ab81289, Abcam),

rat anti-Ly6G (1:300 MA1-70099, Invitrogen).

**Primers for qPCR**

Primers (5’-3’)

GADPH Forward TGAAGGTCGGTGTGAACGG

GADPH Reserve CGTGAGTGGAGTCATACTGGAA

adenosine A1R Forward TGTGCCCGGAAATGTACTGG

adenosine A1R Reserve TCTGTGGCCCAATGTTGATAAG

adenosine A2aR Forward GCCATCCCATTCGCCATCA

adenosine A2aR Reserve GCAATAGCCAAGAGGCTGAAGA

CD16 Forward TTTGGACACCCAGATGTTTCAG

CD16 Reserve GTCTTCCTTGAGCACCTGGATC

IL-17a Forward TTTAACTCCCTTGGCGCAAAA

IL-17a Reserve CTTTCCCTCCGCATTGACAC

iNOS Forward CAAGCACCTTGGAAGAGGAG

iNOS Reserve AAGGCCAAACACAGCATACC

CD206 Forward CAAGGAAGGTTGGCATTTGT

CD206 Reserve CCTTTCAGTCCTTTGCAAGC

IL-10 Forward CTTACTGACTGGCATGAGGATCA

IL-10 Reserve GCAGCTGTAGGAGCATGTGG

TGF-β Forward TGCGCTTGCAGAGATTAAAA

TGF-β Reserve CGTCAAAAGACAGCCACTCA

IL-1β Forward CACCTCTCAAGCAGAGCACAG

IL-1β Reserve GGGTTCCATGGTGAAGTCAAC

TNF-α Forward AAATGGGCTCCCTCTCATCAGTTC

TNF-α Reserve TCTGCTTGGTGGTTTGCTACGAC

CCL-3 Forward TGTACCATGACATCTGCAAC

CCL-3 Reserve CAACGATGAATTGGCGTGGAA


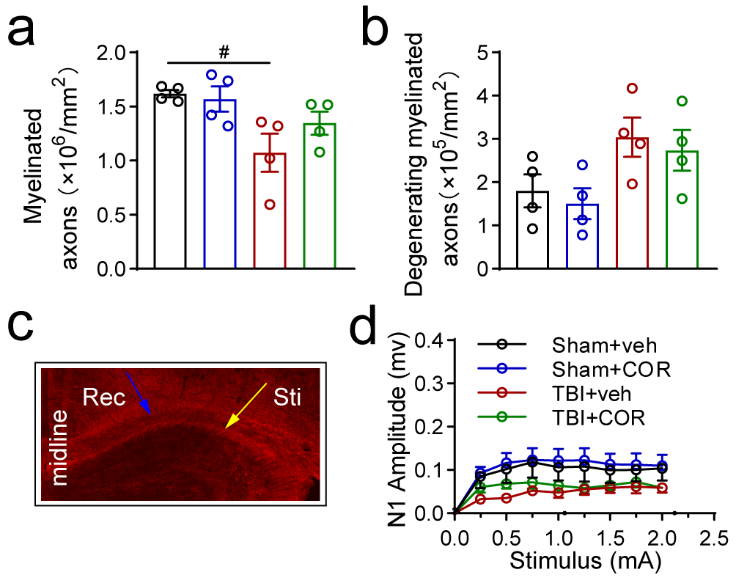


**Supplementary Figure 1 Effect of cordycepin on myelinated axons and CAPs N1 peak.**

**a** Quantification of the numbers of myelinated axons. **b** Quantification of the numbers of the degenerating nonmyelinated axons. n=4/group. **c** The locations where recording electrode and stimulus electrode were placed. **d** Quantification of N1 amplitude for each group. n=6/group. #p < 0.05 , as indicated..


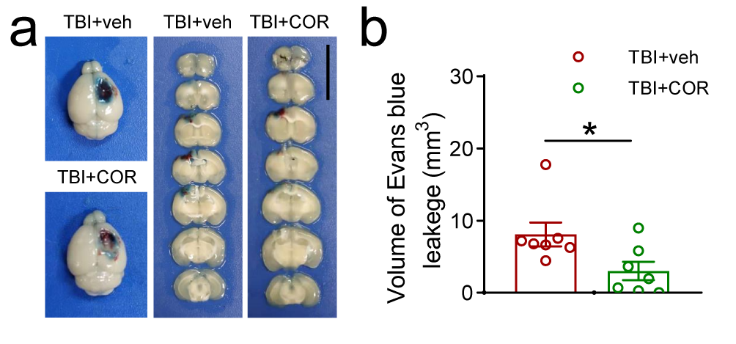


**Supplementary Figure 2 Effect of cordycepin on Evans blue leakage.**

**a** Representative leakage images of each group on 3d after TBI, scale bar: 1 mm. **b** Quantification of Evans blue leakage volume. *p < 0.05, as indicated..


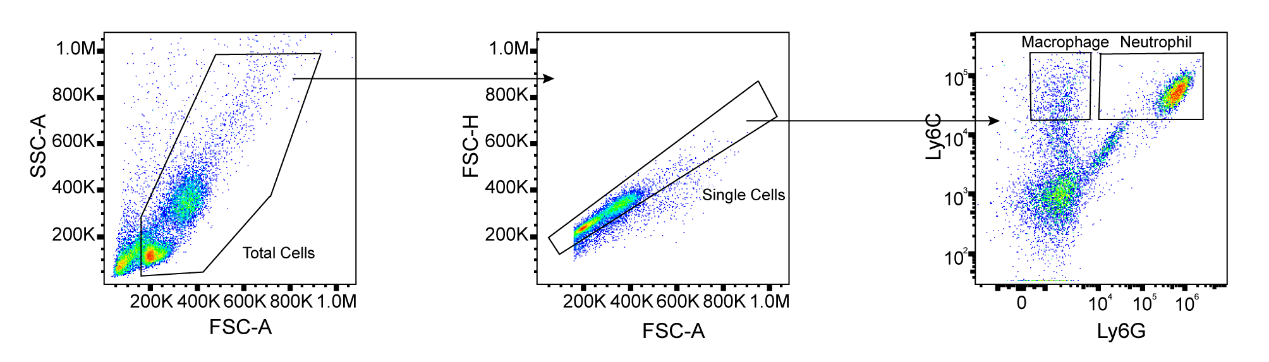


**Supplementary Figure 3 Representative Flow Cytometry analysis of the neutrophil depletion effect in blood.**

Blood was obtained from heart at 48 h after the second injection of Ly6G antibody. Enriched immune cells were stained with anti-Ly6C and anti-Ly6G to distinguish macrophage (Ly6C^high^Ly6G^-^) and neutrophil (Ly6C^high^Ly6G^+^).
